# Individual and combined effects of the infrared, visible, and ultraviolet light components of solar radiation on damage biomarkers in human skin cells

**DOI:** 10.1096/fj.201902351RR

**Published:** 2020-01-16

**Authors:** Laura Hudson, Eyman Rashdan, Catherine A. Bonn, Bhaven Chavan, David Rawlings, Mark A. Birch‐Machin

**Affiliations:** ^1^ Dermatological Sciences Translational and Clinical Research Institute Medical School Newcastle University Newcastle upon Tyne NE2 4HH UK; ^2^ Croda Europe Ltd Snaith UK; ^3^ Northern Medical Physics and Clinical Engineering Freeman Hospital Newcastle upon Tyne UK

**Keywords:** oxidative stress, photoaging, photodamage, photoprotection, sunlight

## Abstract

The ability of solar ultraviolet (UV) to induce skin cancer and photoaging is well recognized. The effect of the infrared (IR) and visible light (Vis) components of solar radiation on skin and their interaction with UV is less well known. This study compared the effects of physiologically relevant doses of complete (UV + Vis + IR) solar‐simulated light and its individual components on matched primary dermal fibroblasts and epidermal keratinocytes from human donors on three biomarkers of cellular damage (reactive oxygen species (ROS) generation, mitochondrial DNA (mtDNA), and nuclear DNA (nDNA) damage). There was a greater induction of ROS, mtDNA, and nDNA damage with the inclusion of the visible and IR components of solar‐simulated light in primary fibroblast cells compared to primary keratinocytes (*P* < .001). Experiments using exposure to specific components of solar light alone or in combination showed that the UV, Vis, and IR components of solar light synergistically increased ROS generation in primary fibroblasts but not primary keratinocytes (*P* < .001). Skin cell lines were used to confirm these findings. These observations have important implications for different skin cell type responses to the individual and interacting components of solar light and therefore photodamage mechanisms and photoprotection interventions.

**Abbreviations**
COX: cytochrome C oxidase
ECM: extracellular matrix
ETC: electron transport chain
H_2_O_2_: hydrogen peroxide
HaCaT: human keratinocyte cell line
HDFn: human dermal fibroblast (neonatal)
IR: infrared light, 780‐3000 nm
MMP: matrix metalloproteinase
mtDNA: mitochondrial DNA
nDNA: nuclear DNA
qPCR: real time quantitative PCR
ROS: reactive oxygen species
SED: standard erythemal dose
UV: ultraviolet light, 290‐400 nm
UVA: ultraviolet A light, 320‐400 nm
UVB: ultraviolet B light, 290‐320 nm
Vis: visible light, 400‐780 nm

## 1 INTRODUCTION

The damaging effects of excessive ultraviolet radiation (UV) on skin following overexposure to sunlight are well characterized by skin reddening, blistering and burning, leading to accelerated aging, and increased susceptibility to skin cancer.^1^, ^2^ Until recently, this has been thought to be caused by only UV light, which accounts for approximately 6.8% of the solar radiation reaching the Earth's surface. Although UV is the higher energy wavelength, the remaining 93.2% consists of the longer wavelength infrared radiation (IR) and visible light (Vis) bands, both of which until recently have been less well studied.^3^, ^4^, ^5^ IR exhibits a number of biological effects, most notably the increase in matrix metalloproteinase (MMP) mRNA and protein expression levels, contributing to the aging phenotype observed in skin.^6^, ^7^ The reported effects of IR can be variable depending on the dose and pattern of IR application.^8^ Similarly, the skin's response to Vis is less well documented although Vis‐induced reactive oxygen species (ROS) generation and skin tanning have been reported.^9^, ^10^, ^11^ As solar radiation is polychromatic,^4^, ^12^ the interaction of all three individual components (UV, IR, and Vis) as well as the effects of combinations of the components warrants further investigation.

In addition, the effects of these wavelength components on the predominant skin cell types, namely keratinocytes and fibroblasts, should be also be considered. The skin consists of layers, which contain different cell types.^13^ The outermost is the stratum corneum, which provides a barrier against external insults (eg, mechanical damage, bacteria, heat etc). The layer below is the epidermis where keratinocyte cells proliferate to constitute the stratum corneum. Fibroblasts are the primary cell type in the dermis, which lies underneath the epidermis, and their primary role is to maintain the extracellular matrix (ECM), which contains collagen and elastin and provides skin structure. When stressed by factors such as UV light, they can dysregulate the ECM, breaking down collagen and elastin, leading to wrinkling and premature skin aging.^13^

Cellular ROS have important roles in cell signaling and homeostasis. They are formed as a natural by‐product of the normal metabolism of oxygen, predominantly (90%) in the mitochondria.^14^, ^15^ Following exposure to environmental stress (eg, UV and environmental pollution), increased ROS levels lead to cell structure damage due to oxidative stress.^16^ Hydrogen peroxide (H_2_O_2_) is one of many types of ROS produced under normal circumstances and is increased as a result of external stressors such as sunlight. Its formation is linked to other forms of ROS in cells, and has been associated with cellular senescence.^17^ There are multiple copies of mitochondrial DNA (mtDNA) within each organelle and the genome is found in close proximity to the site of ROS production, therefore making mtDNA vulnerable to damage by ROS.^18^ Mitochondrial DNA damage leads to further mitochondrial dysfunction and ROS production (increasing oxidative stress within the cell) leading to a putative cycle of ROS production and associated mitochondrial damage.^14^, ^18^ mtDNA damage has been implicated in the aging process in several organs, especially the skin, and mtDNA as a biomarker of damage has been previously demonstrated to reliably and sensitively detect UV‐induced cellular damage.^1^ Furthermore, nuclear DNA (nDNA) acts as a chromophore primarily for ultraviolet B (UVB, 290‐320 nm) leading to increased photoproducts^19^, ^20^, ^21^, ^22^, ^23^ and nDNA (as well as mtDNA) is damaged indirectly by longer wavelength ultraviolet A (UVA, 320‐400 nm) induced ROS.

This study aims to compare the effects of complete and IR/Vis filtered solar‐simulated light on human primary dermal skin fibroblasts and matched epidermal keratinocytes from different donors on the three biomarkers of cellular damage described above, namely ROS generation, mtDNA and nDNA damage. Skin cells were exposed to physiologically relevant doses of complete solar light comprising of UV, IR, and Vis and specific filters were used to investigate exposure to those specific components of solar light either alone or in combination. nDNA damage was measured by comet assay; mtDNA damage was measured by real time quantitative PCR (qPCR) and H_2_O_2_ generation was measured by a luminescence based assay as an indicator of ROS production Established cell lines, human neonatal dermal fibroblast (HDFn) and the immortalized human skin keratinocyte (HaCaT) cells were used to confirm findings where appropriate.

## 2 MATERIALS AND METHODS

### 2.1 Cell culture

Human primary skin fibroblasts and keratinocytes were cultured from healthy adult skin obtained from donors from the Royal Victoria Infirmary and Newcastle Freeman Hospital (Newcastle upon Tyne, United Kingdom). All human tissue work adhered to the guidelines outlined by the Newcastle and North Tyneside Research Ethics Committee (Ref 08/H0906/95 + 5), and Newcastle upon Tyne Hospitals NHS Foundation Trust. The study adhered to the Declaration of Helsinki principles. Human primary keratinocytes were grown in Epilife Medium (Life Technologies, Paisley, UK) with human keratinocyte growth supplement (Life Technologies, Paisley, UK). Human primary fibroblasts, neonatal dermal fibroblast cell line (HDFn) (Invitrogen), and the immortalized human skin keratinocyte cell line (HaCaT)^24^ were grown in DMEM containing 10% of fetal calf serum and 1% penicillin/streptomycin (Lonza) in a humidified atmosphere with 5% of CO_2_ at 37°C. All cell lines were tested for mycoplasma every 3 months.

### 2.2 ROS‐Glo assay—cellular ROS generation

The ROS‐GloTM H_2_O_2_ assay (Promega) was used to assess cellular H_2_O_2_ levels according to the manufacturer's instructions. The plate was read using a Glo‐Max luminometer with the Cell‐titre Glo in built protocol (PMT activated). Menadione (20 μM) was used as a positive experimental control.

### 2.3 Real Time‐QPCR mtDNA strand break assay

Mitochondrial DNA damage was quantified using an 11 kb long‐range qPCR methodology that sensitively detects mtDNA strand breaks, as only undamaged DNA is amplified. Short mitochondrial and nuclear DNA housekeeping amplicons (83 bp and 93 bp, respectively) control for any variation in mtDNA copy number.^25^ The change in cycle threshold (ct) of the irradiated samples compared to the unirradiated samples is expressed as a fold change. qPCR amplification of the 11 kb mtDNA region was carried out on the StepOnePlusTM machine (Applied Biosystems). Samples from each experiment were performed in triplicate for each condition. A positive control of known cycle threshold value was included with each reaction along with a negative control containing master mix only. Analysis was performed using the StepOnePlusTM v2.3 software. The correct product sizes were assessed using melt curve analysis.

### 2.4 Comet assay

The comet assay was performed as described previously by Oyewole et al, 2014.^26^ Nucleoids were analyzed using Comet assay ΙV software (Perceptive instruments, UK) in order to determine the mean tail length of each treatment. The tail length (measure of DNA migration from the head of the comet) was measured and normalized against the control (untreated) sample. An increase in nuclear DNA damage results in increased strand breaks which is reflected by an increase in length of the comet tail. One hundred nucleoides chosen at random were measured per slide.

### 2.5 Solar light sources and filters

The Newport solar simulator (Class ABA) containing a xenon arc lamp was used to provide controlled illumination consisting of UV (280‐400 nm), Vis (400‐780 nm) and IR (780‐3000 nm). One standard erythemal dose (SED) would typically be delivered in 1 minute and be equivalent to 1 hour in the Mediterranean, June, noon sunlight. An SED is a unit of erythemally weighted radiant exposure equivalent to 100 Jm^−2^.^27^ The lamp was calibrated annually from 200‐700 nm using a Bentham DMc150 Double Monochromator (Bentham Instruments Limited, UK). The exact time to irradiate with 2.16 SEDs was calculated before each experiment from a reading with an ILT1400 radiometer as the time for 100 Jm^−2^ of erythemally weighted UV to be emitted with the IR/Vis filter present. This time was then used for all filter conditions in that technical repeat. To irradiate with higher doses, the irradiation time was increased accordingly.

**Figure 1:**
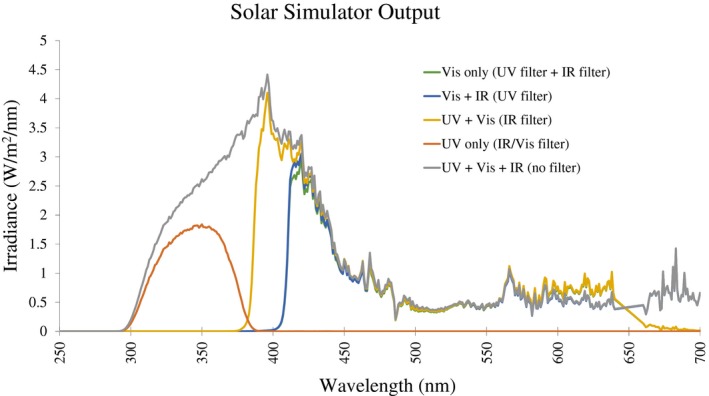
Output from Newport Solar Simulator, measured between 250‐700 nm with and without filters to isolate components of solar light. Measurement and calibration of lamp by the Newcastle Regional Medical Physics Department (Mr David Rawlings)

The IR/Vis filter (UG11 Glass‐Type, UQG optics, Cambridge, UK) band‐pass filter permits passage of UV while blocking the IR and Vis regions. The IR cutoff and UV blocking filters were both purchased from UQG Optics. The IR filter also blocks UV below 380 nm, meaning that the “UV + VIS” condition (in parts of Figures 4 and 5) contains only a small proportion of UV compared to the UV + VIS + IR and UV‐only conditions. However, as some UV remains when using the IR filter, this is indicated in the condition name as UV + VIS as opposed to simply VIS. Figure 1 shows the profile from the solar simulator using either single or combinational use of these filters to produce UV, Vis, or IR wavelength combinations.

### 2.6 Statistical analysis

One‐ and two‐way ANOVAs with correction for multiple groups and two‐tailed, unpaired *t* tests (see figure legends) were performed using commercially available software (GraphPad Prism 5; GraphPad, San Diego, CA, USA).

## 3 RESULTS

To compare the effects of complete and IR/Vis filtered solar‐simulated light on primary dermal fibroblasts and epidermal keratinocytes from different donors, three biomarkers of cellular damage were used, namely ROS generation, mtDNA, and nuclear damage as detailed in the methods.

### 3.1 Visible and IR components of solar‐simulated light increase biomarkers of UV damage in fibroblasts but not keratinocytes

**Figure 2:**
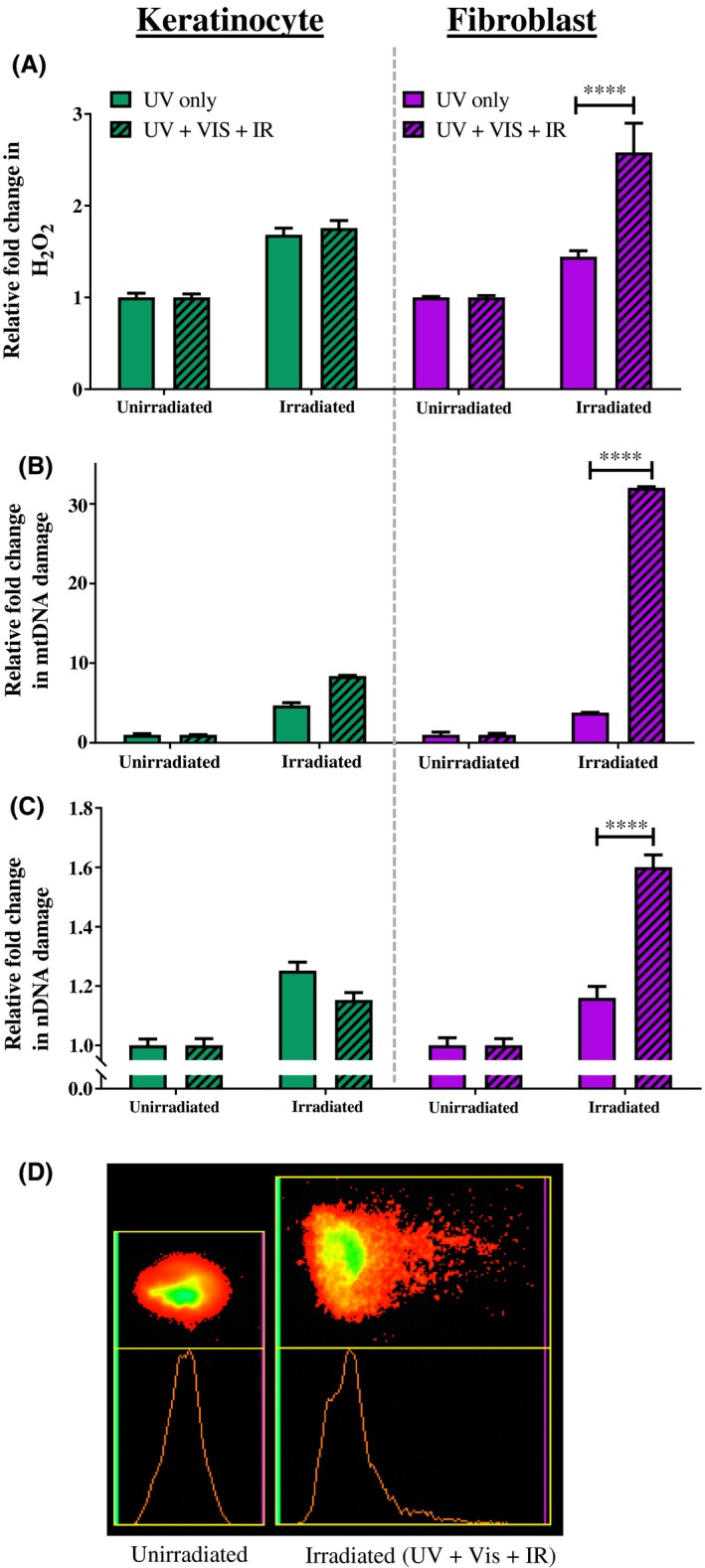
Increased cellular damage markers observed in primary cells exposed to complete solar‐simulated light (UV + Vis + IR) compared to UV alone. Cells were irradiated with 2.16 SED compared to unirradiated control. A, ROS‐Glo assay. B, mtDNA damage qPCR assay. C, nDNA damage comet assay. D, Comet assay image—shows spread of nuclear DNA of unirradiated cell nucleus (left) and after damage from complete solar‐simulated light (right). Error bars represent means SEM (N = 3). Statistical significance was assessed by performing an ANOVA with Bonferroni correction with multiple groups. ****P < .0001

First, the effect of filtered solar‐simulated UV light (ie, UV‐only, without the presence of visible and IR light) on the cellular biomarkers was investigated. Donor‐matched primary dermal fibroblasts and keratinocytes were irradiated with a 2.16 SED dose that is equivalent to 2 hours of Mediterranean sun. Compared to unirradiated controls, UV‐only caused similar increases in ROS in both cell types (Figure 2A) (68% in keratinocytes and 44% in fibroblasts, no significant difference between these increases, *P* > .9999).

Unlike the response to UV‐only filtered solar‐simulated light, the response of the biomarkers to complete (unfiltered) solar‐simulated light (UV + VIS + IR) differs between the two cell types. In primary keratinocytes, complete solar‐simulated light increased H_2_O_2_ to a similar extent to UV‐only light (1.76‐fold increase vs 1.68‐fold increase, respectively, no significant difference). In contrast, primary fibroblasts had a significantly greater increase in ROS in response to complete solar‐simulated light than to UV alone (2.58‐fold increase vs 1.44‐fold increase, respectively, *P* < .0001). These experiments were repeated at a quarter of the dose (ie, 0.54 SED). At this lower dose, the trend of damage was the same as that seen for 2.16 SEDs, but the effects were not significant as the magnitude of each response was much lower (data not shown).

The pattern of induced mtDNA damage observed following the two conditions of irradiation mirrored that observed for H_2_O_2_ generation in each skin cell type (Figure 2B). In detail, both cell types exhibit increased mtDNA damage from UV‐only light compared to unirradiated controls (4.7‐fold in keratinocytes and 3.8‐fold in fibroblasts, *P* < .0001). While both cell types exhibited further increased mtDNA damage induced by complete solar light (UV + VIS + IR) compared to UV‐only, the damage in the fibroblasts was significantly higher than that in keratinocytes (32‐fold higher damage vs 8.4‐fold greater damage, *P* < .0001).

Nuclear DNA damage (Figure 2C) increased in a similar pattern as that observed for H_2_O_2_ generation and mtDNA damage in Figure 2, parts A and B. Both UV‐only and complete solar light significantly increased nDNA damage in fibroblasts and keratinocytes (*P* < .001). The damage from complete solar light was greater in fibroblasts than keratinocytes (1.6‐fold vs 1.15‐fold, *P* < .0001), and in fibroblasts, the response to complete solar light was significantly greater than the response to UV‐only light (1.6‐fold vs 1.16‐fold, *P* < .0001).

In each cell type, both conditions (UV‐only and complete solar light) caused significant increases in all three biomarkers compared to unirradiated controls (*P* ≤ .0009, except for the single example of fibroblasts in Figure 2A in the UV‐only condition where the increase was close to significance, namely *P* = .0525).

### 3.2 Cell lines replicate the pattern of induced biomarker response observed in primary cells

**Figure 3:**
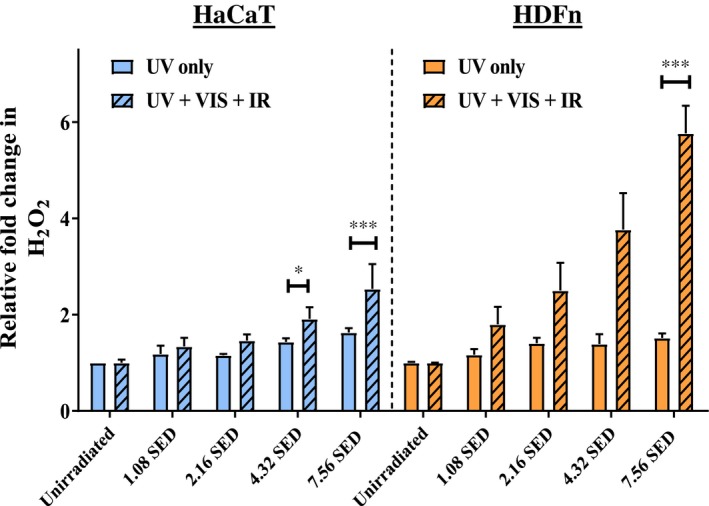
Cell line fibroblast (HDFn) and keratinocyte (HaCaT) responses to increasing doses of light. Error bars represent SEM (N = 3 for HDFn, N = 2 for HaCaT). Statistical significance was assessed by performing an ANOVA with Bonferroni correction for multiple comparisons. *P < .05, ***P < .001

To confirm the observations in primary cells, the experiment described in Figure 2A was repeated for a human fibroblast (HDFn) and keratinocyte (HaCaT) cell line but using a greater range of solar light doses (1.08‐7.56 SED, equivalent to 1‐7 hours in the midday Mediterranean sun). The HDFn and HaCaT cell lines exhibited the same pattern of ROS generation as their counterpart primary cells (Figure 3). The HDFn and HaCaT cell lines were similarly affected by UV light alone in terms of H_2_O_2_ generation (no significant difference) and the HDFn cells had greater H_2_O_2_ production than HaCaTs in response to complete solar light. This pattern continued up to the highest dose tested, 7.56 SED, where there was a 5.8‐fold increase in H_2_O_2_ for HDFn fibroblasts and a 2.5‐fold increase for HaCaT keratinocytes (*P* < .001).

### 3.3 Putative wavelength synergy in H_2_O_2_ ROS generation observed in primary fibroblasts, but not keratinocytes

**Figure 4:**
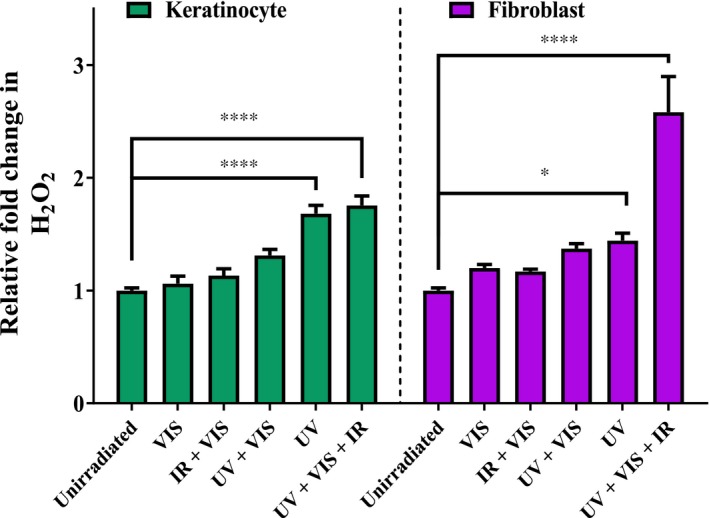
Putative synergy of H_2_O_2_ induction by complete solar light (UV + Vis + IR) compared to its individual components observed in human primary fibroblasts but not primary keratinocytes. Cells were irradiated with 2.16 SED solar‐simulated light and hydrogen peroxide generated was detected by the ROS‐Glo assay. The UV + VIS condition contains only UV above 380 nm (see methods). Error bars represent means SEM (N = 3). Statistical significance was assessed by performing a two‐way ANOVA with Bonferroni correction with multiple groups. *P < .05, ****P < .0001

The results described above suggest that the longer wavelengths of visible and infrared appear to induce a greater cellular damage response in fibroblasts when compared to keratinocytes. This observation was further investigated by utilizing exposure to specific components of solar light and those components in combination. The UV, visible, and IR components of solar light were found to synergistically increase H_2_O_2_ generation in primary fibroblasts but not primary keratinocytes (Figure 4). These individual components of solar light and combinations thereof were examined to test their contributions to H_2_O_2_ generation in primary skin cells. In terms of H_2_O_2_ generation, keratinocytes, and fibroblasts responded similarly (no significant difference) in all irradiation conditions apart from complete solar‐simulated light irradiation (UV + Vis + IR), where fibroblasts had a significantly greater response (ie, 2.6‐fold (fibroblasts) increase compared to unirradiated vs 1.8‐fold (keratinocytes), *P* < .0001). This response observed in fibroblasts appeared to be synergistic, in that complete solar‐simulated light caused a greater increase in H_2_O_2_ generation than the sum of its component parts.

### 3.4 Putative wavelength synergy in H_2_O_2_ ROS generation observed in a fibroblast cell line

**Figure 5:**
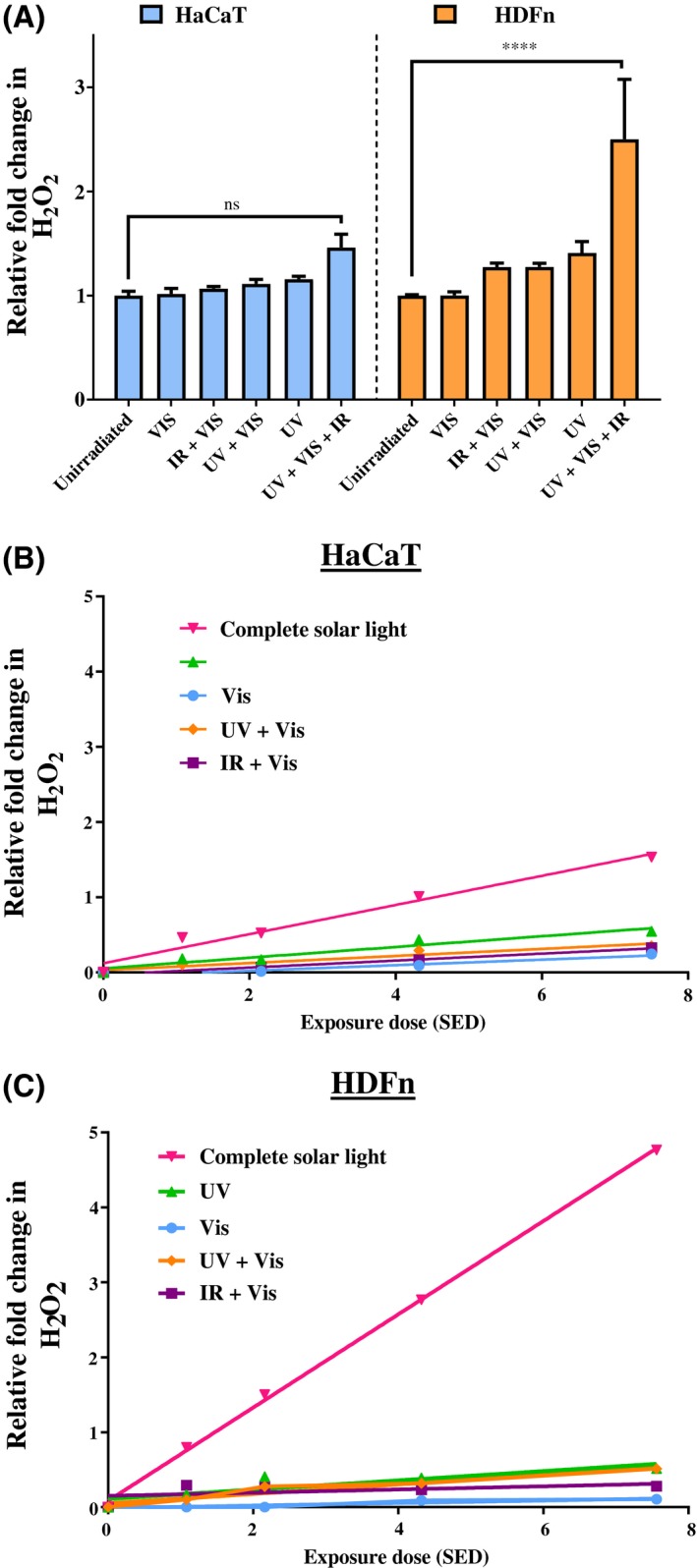
A, Putative synergy of ROS production by complete solar‐simulated light (UV + Vis + IR) compared to solar UV alone observed in the cell line dermal fibroblasts (orange) but not epidermal keratinocytes (blue). The UV + VIS condition contains only UV above 380 nm (see methods). Cell lines (HaCat—keratinocyte and HDFn—fibroblast) irradiated with components of 2.16 SED solar‐simulated light, H_2_O_2_ detected by ROS‐Glo assay. Error bars represent means SEM (N = 3 for HDFn, N = 2 for HaCaT). Statistical significance was assessed by performing a two‐way ANOVA with Bonferroni correction for multiple comparisons (****P < .0001). B, HaCaT keratinocyte cell line responses to components of solar light at increasing doses. C. HDFn fibroblast cell line responses to components of solar light at increasing doses

The series of experiments described above for Figure 4 was repeated in the HDFn human fibroblast and HaCaT human keratinocyte cell lines. Both cell lines were exposed to the same regime of UV, visible, and IR components of solar‐simulated light and were found to respond in a similar pattern as primary cells (Figure 5A) in terms of induced H_2_O_2_ generation. Compared to the primary dermal fibroblast cells, HDFn cells had the same pattern of increased H_2_O_2_ with compete solar‐simulated light (UV + Vis + IR) (no significant difference, two‐way ANOVA with Bonferroni correction of data from Figure 4 vs 5A). Compared to the primary keratinocytes, the HaCaT keratinocyte cell line had a similar pattern of response to all components and combinations, though in the HaCaT cells the increase in H_2_O_2_ was not formally significant (Figure 5A, *P* = .8336). The HFDn fibroblast cell line had a greater response of ROS generation induced by complete solar‐simulated light than the HaCaT cells by a factor of 104% (*P* < .01). These results indicate that the apparent wavelength synergy of ROS generation observed in primary cells is also exhibited by these human cell lines and that the effects observed in primary cells are not an artifact of donor variability.

In order to determine the effects of dose on H_2_O_2_ response to components of solar‐simulated light, cell line keratinocytes and fibroblasts were irradiated with increasing doses of UV, Vis, and IR and their combinations (Figure 5B and 5 respectively). For all conditions, a linear effect of dose on H_2_O_2_ generation was observed in both cell types. The apparent synergistic effect of solar light components on the HDFn fibroblasts can be clearly seen at all doses of solar light from 1.08 SED to 7.56 SED. When the slopes of the lines of best fit of H_2_O_2_ generation between the fibroblasts and keratinocytes were compared, the complete solar light (UV + Vis + IR) condition had a significantly different slope (*P* < .0001, two‐tailed ANCOVA), but for other conditions there were no significant differences. If the increases in H_2_O_2_ following a dose of 7.56 SED of solar‐simulated light are taken as an example, one can identify a putative synergistic effect of the combined wavelengths of UV, Vis and IR in the complete solar light (Table 1). The data in Table 1 shows, particularly in fibroblasts, that the induced responses to the individual light components do not sum to the total induced response of the cells to these components in complete solar light (UV + Vis + IR).

**Table 1:** Increases in H_2_O_2_ generation in cell line skin cells following a 7.56 SED solar‐simulated dose equivalent to 7 hours of midday June Mediterranean sunlight

	Increase in H_2_O_2_ ROS generation by 7.56 SED complete solar light (% increase compared to unirradiated control)				
	Visible	IR + Visible	UV + Visible	UV	UV + Visible + IR
HaCaT	24	33	35	55	153
HDFn	11	28	51	52	476

Note

The putative synergy of light components in the UV + Vis + IR condition is particularly obvious in the HDFn fibroblast cell line condition. The data are derived from the graphs in Figure 5B,C.

## 4 DISCUSSION

This study compared the effects of complete solar light containing UV, Vis, and IR lights versus IR/Vis filtered (UV‐only) solar‐simulated light on primary dermal fibroblasts and epidermal keratinocytes from different donors on three biomarkers of cellular damage, namely ROS generation, mtDNA damage, and nDNA damage. nDNA damage was measured by comet assay; mtDNA damage was measured by real time quantitative PCR and hydrogen peroxide (H_2_O_2_) generation was measured by a luminescence based assay as an indicator of ROS production. The study found that under the experimental conditions (ie, complete solar‐simulated light vs components of solar‐simulated light under different filtered conditions), there is a greater induction of ROS, mtDNA, and nDNA damage by the visible and IR components of solar‐simulated light in primary fibroblast cells compared to primary keratinocytes. The use of a filter to remove the IR and Vis wavelengths (thereby leaving UVA and UVB) had no detectable effect on damage in keratinocytes as levels of ROS, nDNA damage and mtDNA damage were similar both in the presence and absence of IR and Vis. In contrast, removal of IR/Vis significantly reduced the amount of damage in fibroblasts. As the UV alone component of solar‐simulated light affected both cell types similarly, it appears that there is some aspect of wavelength synergy evidenced in the additional biomarker damage induced by the Vis and IR light components on fibroblasts (but no additional damaging effects in keratinocytes). These differential responses between fibroblasts and keratinocytes were also observed in both human skin keratinocyte and fibroblast cell lines (HaCaT and HDFn, respectively) thereby suggesting that the effects observed in primary cells are not an artifact of donor variability.

Our work confirms but also extends observations in previous studies. For example, our previous study^28^ which determined the UV action spectrum of mtDNA damage in different skin cell types showed a greater induction of mtDNA damage at UV wavelengths greater than 300 nm in fibroblasts compared to keratinocytes. Our current work extends this investigation into the Vis and IR range, using three biomarkers as opposed to one (ie, mtDNA damage) and using not only primary human skin cells but also matched keratinocytes and fibroblasts from the same donors. Differential responses to UV between keratinocytes and fibroblasts have also been observed by Derrico et al, 2007^29^ who reported that keratinocytes were more resistant than fibroblasts to the lethal effects of UV and more efficient in the removal of cyclobutane pyrimidine dimers. In addition, Bernerd and Asselineau, 1998^30^ suggested differential cell type sensitivity (apoptosis and tissue repair) to UVA demonstrating that dermal fibroblasts are less resistant to UVA.

The comparative resistance of keratinocytes to damage by solar wavelengths longer than UV (ie, IR and Vis) may be due partly to their higher anti‐oxidative capacity compared to that observed in fibroblasts.^31^ Interestingly in this respect, IR is thought to elicit a retrograde signaling response involving ROS, which itself is initiated in the mitochondrial electron transport chain (ETC).^32^ Compared to fibroblasts, keratinocytes have a reported two‐ to fivefold lower ETC activity which is directly related to superoxide ROS production following leakage of electrons combining with molecular oxygen.^33^ Furthermore, IR is mainly absorbed by the cytochrome C oxidase (COX) components that also serve as a site of ROS generation and therefore a lower ETC activity in keratinocytes compared to fibroblasts could reduce the overall IR effect on COX. Keratinocytes also contain higher levels of ferritin than fibroblasts which would provide a greater protective effect against oxidative stress by chelating iron which might otherwise catalyze the formation of damaging hydroxyl radicals induced by IR.^34^ It is likely to be a combination of the above scenarios working together which would account for the increased resistance and/or decreased response to IR and Vis in keratinocytes compared to fibroblasts.^8^, ^28^, ^31^ However, it is clear that decreased ROS production in keratinocytes compared to fibroblasts would clearly lead to a decreased burden on DNA damage (both mitochondrial and nuclear)^26^, ^35^ as observed in our study. Although there are relatively few studies using Vis wavelengths, Liebel et al, 2012^36^ have suggested that Vis‐induced ROS is produced in human skin equivalents^37^, ^38^ although there is no comment on differential responses in cell types.

Our current study went further using filters to investigate exposure to specific components of solar light and those components in combination. In summary, the UV, Vis, and IR components of solar light appear to increase ROS generation in a synergistic manner in primary fibroblasts but not primary keratinocytes. Solar radiation is polychromatic and its effects on skin are not only the result of the separate action of each wavelength but rather the result of the interaction of the numerous wavelengths.^12^ UV, IR, and Vis target different chromophores within the skin leading to increased ROS. UV is absorbed mainly by DNA, aromatic amino acids, and other chromophores such as riboflavin, while IR is absorbed predominantly by components in the mitochondrial COX complex. Less is known about the chromophores for Vis, however potential chromophores reported include bilirubin, hemoglobin, melanin, and β‐Carotene.^6^, ^36^, ^38^ Although IR, Vis, and UV have all been reported to generate ROS within cells, they do so through different mechanisms. Solar UV, particularly UVA, leads to ROS generation resulting in indirect DNA damage^39^ including both nDNA and mtDNA damage.^7^, ^35^, ^40^ There is evidence of Vis‐induced ROS^36^, ^41^ leading to skin pigmentary effects.^10^ Exposure to IR has been shown to result in the generation of ROS although an indirect action has been proposed to be due to heat generation resulting in the mobilization of heat stressor proteins and heat sensors promoting the inward cellular flux of calcium also leading downstream to MMP induction.^6^, ^42^, ^43^ However the relevance to normal physiological conditions in sunlight is unclear.

The observations in our study have important implications for photodamage mechanisms and related photoprotection interventions together with a skin cell type sensitivity to the individual and interacting components of solar light including UV, Vis, and IR.

## CONFLICT OF INTEREST

The authors declare no conflicts of interest.

## AUTHOR CONTRIBUTIONS

M.A. Birch‐Machin as the senior and corresponding author co‐designed the research with B. Chaven; D. Rawlings performed the lamp calibrations and provided photobiology advice; L. Hudson and E. Rashdan performed the research; L. Hudson, E. Rashdan, and C.A. Bonn analyzed data; E. Rashdan, C.A. Bonn, and M.A. Birch‐Machin wrote the paper.
